# Genome-wide associations and detection of potential candidate genes for direct genetic and maternal genetic effects influencing dairy cattle body weight at different ages

**DOI:** 10.1186/s12711-018-0444-4

**Published:** 2019-02-06

**Authors:** Tong Yin, Sven König

**Affiliations:** Institute of Animal Breeding and Genetics, Justus-Liebig-University Gießen, Ludwigstr. 21b, 35390 Giessen, Germany

## Abstract

**Background:**

Body weight (BW) at different ages are of increasing importance in dairy cattle breeding schemes, because of their strong correlation with energy efficiency traits, and their impact on cow health, longevity and farm economy. In total, 15,921 dairy cattle from 56 large-scale test-herds with BW records were genotyped for 45,613 single nucleotide polymorphisms (SNPs). This dataset was used for genome-wide association studies (GWAS), in order to localize potential candidate genes for direct and maternal genetic effects on BW recorded at birth (BW0), at 2 to 3 months of age (BW23), and at 13 to 14 months of age (BW1314).

**Results:**

The first 20 principal components (PC) of the genomic relationship matrix (${\mathbf{G}}$) grouped the genotyped cattle into three clusters. In the statistical models used for GWAS, correction for population structure was done by including polygenic effects with various genetic similarity matrices, such as the pedigree-based relationship matrix (${\mathbf{A}}$), the ${\mathbf{G}}$**-**matrix, the reduced ${\mathbf{G}}$**-**matrix LOCO (i.e. exclusion of the chromosome on which the candidate SNP is located), and LOCO plus chromosome-wide PC. Inflation factors for direct genetic effects using ${\mathbf{A}}$ and LOCO were larger than 1.17. For ${\mathbf{G}}$ and LOCO plus chromosome-wide PC, inflation factors were very close to 1.0. According to Bonferroni correction, ten, two and seven significant SNPs were detected for the direct genetic effect on BW0, BW23, and BW1314, respectively. Seventy-six candidate genes contributed to direct genetic effects on BW with four involved in growth and developmental processes: *FGF6*, *FGF23*, *TNNT3*, and *OMD*. For maternal genetic effects on BW0, only three significant SNPs (according to Bonferroni correction), and four potential candidate genes, were identified. The most significant SNP on chromosome 19 explained only 0.14% of the maternal de-regressed proof variance for BW0.

**Conclusions:**

For correction of population structure in GWAS, we suggest a statistical model that considers LOCO plus chromosome-wide PC. Regarding direct genetic effects, several SNPs had a significant effect on BW at different ages, and only two SNPs on chromosome 5 had a significant effect on all three BW traits. Thus, different potential candidate genes regulate BW at different ages. Maternal genetic effects followed an infinitesimal model.

**Electronic supplementary material:**

The online version of this article (10.1186/s12711-018-0444-4) contains supplementary material, which is available to authorized users.

## Background

Some countries with pasture-based production systems consider dairy cow live weight in overall breeding goals or in selection indices [1, 2]. Positive genetic correlations of body weight (BW) with milk yield and protein yield have been reported [3–5]. Feed efficiency reflects the ability of dairy cows to produce more milk for a given feed consumption [6]. Different traits are defined to measure feed efficiency, e.g. the ratio of milk to body weight, feed intake, residual feed intake [7], and feed saved [8]. Most of these definitions imply that BW or changes in BW are taken into account. Moreover, dry matter intake and energy balances are favourably correlated with BW [3]. In addition, BW influences dairy cow fertility and health. For example, survival of new-born calves and calving ease are moderately correlated with birth weight of calves and BW of cows [9]. Berry et al. [5] reported that heavier cows had a shorter interval between calving and first service, but conception rates decreased with increasing BW. In contrast, in heifers, increasing BW was associated with improved non-return rates after 56 and 90 days [4]. Hence, we hypothesize that different genes are involved in BW at different ages, as indicated in quantitative genetic studies via random regression models [4].

On the genomic scale, GWAS for BW or BW indicators have considered only one time point per animal [10–12]. Zhang et al. [12] analysed longitudinal BW records in cattle at 6, 12, 18 and 24 months of age, but BW was predicted from measurements for heart girth and hip height. The aforementioned publications focussed only on the estimation of direct additive genetic effects on BW. However, especially in early life, BW should be separated into direct genetic and maternal genetic effects [13]. Dams with high breeding values for maternal ability provide an improved nourishing environment, with an associated positive impact on survival rates and birth weight in offspring. For a deeper understanding of the mechanisms between direct and maternal effects, it is imperative to detect the functional segments of the genome that contribute to maternal genetic effects on BW, and to study direct-maternal associations on the genomic scale. To date, only a few studies [14–16] have addressed such topics.

The power of GWAS contributes to the detection of significant markers, and, furthermore, has an impact on the identification of associated potential candidate genes. Linkage disequilibrium (LD) is one of the parameters that affects the power of GWAS. The use of a dense 50 K single nucleotide polymorphism (SNP) chip implies that it contains markers that are closely located to the functional mutation and contribute to acceptable LD between markers and causal loci [17]. Body weight is a trait with a moderate to high pedigree-based heritability [4, 5, 18], which is favourable for the detection of QTL. Furthermore, currently, the trend is to use large numbers of female observations for the estimation of SNP effects, which contributes to an increasing number of phenotypic records for GWAS [19], with a positive impact on the statistical power for the detection of SNP effects. Non-causative rare alleles with high frequencies in large half-sib daughter groups might contribute to false positive signals in GWAS. Usually, the first principal components and similarity matrices can be considered in statistical modelling to correct for population stratification [20]. In dairy cattle breeding, deep pedigree information is available, which enables the use of mixed models for GWAS with random polygenic effects based on pedigree [21] or on genomic relationship matrices [22].

Consequently, the objectives of our study were: (1) to perform GWAS using phenotypes and de-regressed proofs for direct genetic and maternal genetic effects on BW at different ages; (2) to correct for population stratification in GWAS when using pedigree-based or genomic relationship matrices, or a combination of relationship matrices with principal components; (3) to infer (co)variance components for/between direct genetic and maternal genetic effects on different scales (pedigree-based genetic parameters, whole genome, and single chromosomes); and (4) to identify associated potential candidate genes for direct genetic and maternal genetic effects.

## Methods

### Phenotype data

Body weight records at birth (BW0), 2 to 3 months of age (BW23), and 13 to 14 months of age (BW1314) were available for 250,173, 42,632 and 54,768 female animals, respectively. The number of animals with phenotypic records at all three age intervals was 15,234. Animals were born between 2004 and 2016, and kept in 56 large-scale dairy cattle test-herds, which were located in the German federal states of Mecklenburg-Westpommerania and Berlin-Brandenburg. For the 250,173 calves, the gestation length of their dams ranged from 265 to 295 days (average: 279.4 days). For BW0, we discarded birth weights above 60 kg or below 20 kg. For the detection of outlier data for BW23 and BW1314, we followed the approach by Yin et al. [4] and calculated studentized residuals and corresponding Bonferroni *p* values (using the outlier test function in the R package “car” [23]). Records were excluded from further analyses when *p* values were lower than 0.05 or higher than 0.95. The pedigree file included 411,943 animals, born between 1948 and 2016.

### Genotype data

Among the Holstein cattle with BW records, 13,827 calves with BW0 records, 4246 calves with BW23 records, and 7920 heifers with BW1314 records, were genotyped. Genotyping was performed using the Illumina Bovine 50 K SNP BeadChip V2 (4120 animals), or the Illumina Bovine Eurogenomics 10 K low-density chip (11,801 animals). Animals with low-density genotypes were imputed to the 50 K chip (according to the routine procedure for official national genetic evaluations [24]). Finally, for all the genotyped cattle, 45,613 SNPs were available that had a call rate higher than 95%, a minor allele frequency higher than 0.01, and did not deviate significantly from Hardy–Weinberg equilibrium (*p* > 0.001). Only SNPs located on *Bos taurus* autosomes (BTA) were considered. Furthermore, we discarded animals with more than 95% identical genotypes. Quality control of SNP data was done by using the GenABEL package in R [25]. In order to verify the impact of LD between SNPs on the inflation factors in GWAS, we applied the indep-pairwise option in PLINK [26]. We eliminated one SNP from pairs of SNPs that had a LD coefficient (*r*^2^) higher than 0.25 [27]. The remaining SNPs after this elimination procedure were defined as pruned SNPs. The numbers of animals, numbers of the full SNPs, and numbers of pruned SNPs, are in Table 1.

**Table 1:** Number of animals and SNPs for the genome-wide association studies

Trait	Dependent variable	#animals^a^	#animals^b^	#markers^c^	#markers^d^
BW0	Phen	13,827	13,714	42,468	11,955
	dDRP	15,921	14,121	42,465	11,954
	mDRP	16,455	16,022	42,540	
BW23	Phen	4246	4219	42,388	11,908
	dDRP	15,921	8017	42,421	11,933
	mDRP	16,455	6803	42,498	
BW1314	Phen	7920	7874	42,443	11,943
	dDRP	15,921	7874	42,443	11,943
	mDRP	16,455	6996	42,503	

BW0: body weight recorded at birth; BW23: body weight recorded at 2 to 3 months of age; BW1314: body weight recorded at 13 to 14 months of age; dDRP: de-regressed proofs for the direct genetic effect; mDRP: de-regressed proofs for the maternal genetic effect

^a^Number of cows with genotypes

^b^Number of cows with genotypes after quality control

^c^Number of markers after quality control

^d^Number of markers after pruning

### Population stratification

The genomic relationship matrix ${\mathbf{G}}$ was constructed as in [28] based on the full SNP dataset, and then used for principal component analysis, in order to visualise possible population stratification for the 15,921 genotyped animals. The software package GCTA [29] generated the first 20 principal components (PC). Then, k-means clustering was applied by including the first 10 PC, because the remaining 10 PC were not informative and overloaded k-means clustering.

### Statistical models

#### Pedigree-based (co)variance components and breeding values

A multiple-trait animal model was defined, in order to infer the genetic components and to estimate breeding values for direct genetic and maternal genetic effects. In this regard, we applied restricted maximum likelihood (REML) via AIREMLf90 from the BLUPF90 software package [30]. The statistical Model 1 for the three BW traits (BW0, BW23, BW1314) in matrix notation was:

1 $${\mathbf{y}} = {\mathbf{Xb}} + {\mathbf{Zu}} + {\mathbf{Wm}} + {\mathbf{Sp}}_{{\mathbf{m}}} + {\mathbf{e}},$$

where ${\mathbf{y}}$ is a vector of phenotypes for BW0, BW23, and BW1314 from 250,173, 42,632 and 54,768 female animals, respectively; ${\mathbf{b}}$ is a vector of fixed effects, including herd, birth year, birth month, and the covariate (linear regression) gestation length for BW0, and age (in days) of the calves/heifers for BW23 and BW1314; ${\mathbf{u}}$ is a vector of direct additive-genetic effects, with ${\mathbf{u}} \sim N\left( {0,{\mathbf{A}}\upsigma_{\text{u}}^{2} } \right)$, where ${\mathbf{A}}$ is the pedigree-based relationship matrix and $\upsigma_{\text{u}}^{2}$ is the direct-genetic variance; ${\mathbf{m}}$ is a vector of random maternal-genetic effects, with ${\mathbf{m}} \sim N\left( {0,{\mathbf{A}}\upsigma_{\text{m}}^{2} } \right)$, where $\upsigma_{\text{m}}^{2}$ is the maternal-genetic variance; ${\mathbf{p}}_{{\mathbf{m}}}$ is a vector of random maternal permanent environmental effects; ${\mathbf{e}}$ is a vector of random residual effects; and ${\mathbf{X}}$, ${\mathbf{Z}}$, ${\mathbf{W}}$, and ${\mathbf{S}}$ are incidence matrices for ${\mathbf{b}}$, ${\mathbf{u}}$, ${\mathbf{m}}$, and ${\mathbf{p}}_{{\mathbf{m}}}$, respectively.

Estimated breeding values from Model 1 for direct genetic and maternal genetic effects were used to calculate de-regressed proofs (DRP) for the direct genetic (dDRP) and for the maternal genetic component (mDRP), respectively, according to Garrick et al. [31]. Only the animals with a DRP weight greater than 0.2 were considered in ongoing GWAS (see Model 3). The number of DRP records for direct genetic and maternal genetic effects is in Table 1. Since animal models generate breeding values for all the animals from the pedigree database, all these animals, including the phenotyped and non-phenotyped animals, were considered for DRP calculations, which means that an increased number of genotyped animals for DRP is available.

#### Genomic heritabilities and correlations

Variance components and correlations for the three BW traits explained by SNPs on all the chromosomes were estimated via genomic REML (GREML), as implemented in GCTA [29]. Model 2 was defined as follows:

2 $${\mathbf{y}} = {\mathbf{Xb}} + {\mathbf{Zu}} + {\mathbf{e}},$$

where, ${\mathbf{y}}$ is a vector of phenotypes for BW0, BW23, and BW1314, and ${\mathbf{b}}$ is a vector of fixed effects including the same effects as specified in Model 1. The variance for additive genetic effects ${\mathbf{u}}$ was equal to ${\text{G}}\upsigma_{\text{u}}^{ 2}$, with ${\text{G}}$ representing the genomic relationship, and $\upsigma_{\text{u}}^{ 2}$ representing the variance explained by SNPs from the full dataset. For the estimation of covariance components in bivariate models, GCTA requires that the fixed effects are the same for both traits. Hence, we ran bivariate models for pairwise combinations of pre-corrected phenotypes for BW0, BW23, and BW1314. The pre-corrected phenotype for a specific genotyped animal was the sum of the estimated direct breeding value, the maternal breeding value, the maternal environmental effect, and the residual (i.e. output from Model 1).

#### Genome-wide association studies

The software package GCTA [29] was also used to estimate SNP effects via linear mixed models with a random polygenic effect. The statistical Model 3 for single marker regression analysis was:

3 $${\mathbf{y}} = {\mathbf{Xb}} + {\mathbf{Wg}} + {\mathbf{Zu}} + {\mathbf{e}},$$

where ${\mathbf{y}}$ is a vector of phenotypes, dDRP or mDRP for BW0, BW23, and BW1314; ${\mathbf{b}}$ is a vector of fixed effects including the same effects as specified in Model 1 for phenotypes as dependent variables, but for DRP, ${\mathbf{b}}$ only considered the overall mean effect; ${\mathbf{g}}$ is the vector for SNP effects; ${\mathbf{u}}$ is a vector of polygenic effects with a variance–covariance structure of ${\mathbf{u}} \sim N\left( {0,{\mathbf{K}}\upsigma_{\text{u}}^{2} } \right)$, where ${\mathbf{K}}$ is the genetic similarity matrix between individuals, and $\upsigma_{\text{u}}^{2}$ is the polygenic variance; ${\mathbf{e}}$ is a vector of random residual effects with ${\mathbf{e}} \sim N\left( {0,{\mathbf{I}}\upsigma_{\text{e}}^{2} } \right)$; and ${\mathbf{X}}$, ${\mathbf{W}}$, and ${\mathbf{Z}}$ are incidence matrices for ${\mathbf{b}}$, ${\mathbf{g}}$, and ${\mathbf{u}}$, respectively. According to the Bonferroni correction, the defined GWAS significant threshold was 0.05/N, where N refers to the number of SNPs. In addition to the Bonferroni correction, a less conservative adjusted *p* value, based on false discovery rate (FDR), was calculated for each SNP [32]. The threshold for FDR significance was 0.05.

The genetic similarity matrix ${\mathbf{K}}$ was constructed with different information sources. First, we created ${\mathbf{K}}$ based on the pedigree relationship matrix ${\mathbf{A}}$, as generated from AIREMLF90. Second, the construction of ${\mathbf{K}}$ was based on the genomic relationship matrix ${\mathbf{G}}$. Due to possible undesired effects of SNP double-counting [33], alternative ${\mathbf{G}}$-matrices excluded all SNPs from the chromosome on which the candidate SNP is located. This strategy is defined as “leave-one-chromosome-out” (LOCO) [34]. Since the length of bovine chromosomes is not constant, many SNPs on the large chromosomes are excluded. Hence, SNPs located on the large chromosomes BTA1 to 11 (these chromosomes contain more than 1500 SNPs) were separated into two segments per chromosome. The modified LOCO approach (LOCO_SEG40, i.e. the 22 segments from chromosomes 1–11 plus the remaining 18 chromosomes) constructed ${\mathbf{G}}$**-**matrices using all SNPs, except those from the respective chromosome segment (for BTA1 to BTA11), or excluding all SNPs from the whole chromosome (for BTA12 to BTA30). In addition, chromosomes were separated into smaller segments according to the number of SNPs with (a) segments including 90–100 SNPs (a total of 441 segments = LOCO_SEG441), and (b) segments including 47–50 SNPs (a total of 864 segments = LOCO_SEG864). In order to account for the loss in similarity due to the deleted chromosome in LOCO, the first 20 PC were included as covariates (LOCO + PC20). However, consideration of 20 PC combined with the LOCO ${\mathbf{G}}$-matrix implies partial overlap of genomic information. Hence, as a further alternative, we focussed on principal component analyses for the ${\mathbf{G}}$**-**matrix from each chromosome, and the first 3, 10 or 20 PC were included as covariates (LOCO + CHR_PC3, LOCO + CHR_PC10, and LOCO + CHR_PC20, respectively). All the similarity matrices (${\mathbf{G}}$**-**matrix, LOCO ${\mathbf{G}}$**-**matrix, and ${\mathbf{G}}$**-**matrix from each chromosome) as mentioned above were constructed based on the full SNP dataset. An additional LOCO scenario using the pruned SNPs (LOCO_prune) was considered, in order to test the effect of LD between SNPs on inflation.

We used the inflation factor ($\uplambda$) as evaluation criterion for the different approaches, which was calculated based on the $\upchi_{i}^{2}$ statistic for the $i$-th SNP:

$$\widehat{\lambda } = \frac{{Median\left( {\upchi_{i}^{2} } \right)}}{0.4549}.$$

The expected inflation factor of value 1 indicates sufficient correction for population stratification. A value above 1.05 indicates inflation in the sample [35], and thus that the detected genome-wide associations might be false positives.

#### Chromosome-wide genomic parameters

Genetic variances for each chromosome were estimated via GREML using the full SNP dataset, and applying GCTA [29]. The univariate Model 4 was:

4 $${\mathbf{y}} = {\mathbf{Xb}} + {\mathbf{Z}}_{1} {\mathbf{u}}_{{\mathbf{i}}} + {\mathbf{Z}}_{2} {\mathbf{u}}_{{{\mathbf{all}}\_{\mathbf{without}}\_{\mathbf{i}}}} + {\mathbf{e}},$$

where ${\mathbf{y}}$ and ${\mathbf{b}}$ are vectors of phenotypes and fixed effects, respectively, as introduced in Model 1; ${\mathbf{u}}_{{\mathbf{i}}}$ is the additive genetic effect with variance of ${\mathbf{G}}_{{\mathbf{i}}}\upsigma_{{{\text{u}}_{\text{i}} }}^{ 2}$, where ${\mathbf{G}}_{{\mathbf{i}}}$ is the genomic relationship matrix constructed from SNPs located on chromosome ${\text{i}}$, and $\upsigma_{{{\text{u}}_{\text{i}} }}^{ 2}$ is the variance explained by SNPs on chromosome ${\text{i}}$; ${\mathbf{u}}_{{{\mathbf{all}}\_{\mathbf{without}}\_{\mathbf{i}}}}$ is the additive genetic effect due to all the SNPs except those on chromosome ${\text{i}}$; ${\mathbf{e}}$ is the residual effect; and ${\mathbf{X}}$, ${\mathbf{Z}}_{1}$, and ${\mathbf{Z}}_{2}$ are incidence matrices for ${\mathbf{b}}$, ${\mathbf{u}}_{{\mathbf{i}}}$ and ${\mathbf{u}}_{{{\mathbf{all}}\_{\mathbf{without}}\_{\mathbf{i}}}}$, respectively. The heritability for each chromosome is equal to the ratio of $\upsigma_{{{\text{u}}_{\text{i}} }}^{ 2}$ divided by the sum including the variance components from all SNPs on chromosome ${\text{i}}$ plus the variance components from all other SNPs plus the residual variance.

### Gene annotation

The database (version UMD3.1) including gene locations, start positions and end sites for all bovine genes was downloaded from Ensembl [36]. Originally, 24,616 gene ID entries were available in the database. However, only the 17,545 genes on BTA1 to 29 with valid evidences for gene ontology [37, 38] were considered in subsequent analyses. First, SNPs used for GWAS (i.e. the full SNP dataset) were mapped to the genes, by applying the MAGMA software [39], and considering a window 100 kb upstream and downstream for each gene. In the next step, a test statistic for each gene was generated by summing − 2log(*p* values) from a set of SNPs within the aforementioned window. This test followed a Chi square distribution [39]. Also, the *p*-value for each of the 17,545 genes was calculated, and further adjusted according to the FDR [32]. Only the genes with a FDR lower than 5% were considered as significantly associated with one of the BW traits. Then, functional classification analyses were conducted for the significant candidate genes, based on information from the PANTHER database [40].

## Results and discussion

### Genetic parameters

Direct pedigree-based heritabilities (i.e. using the ${\mathbf{A}}$-matrix) for BW traits were moderate to high: 0.46 for BW0, 0.37 for BW23, and 0.48 for BW1314 (Table 2). Similar heritabilities were reported in previous quantitative genetic studies for BW [5, 18, 41]. Interestingly, the maternal genetic component had also a moderate contribution, even for BW1314 recorded later in life. Maternal heritabilities of 0.14, 0.11 and 0.13 were found for BW0, BW23, and BW1314, respectively. Although the genomic relationship matrix (${\mathbf{G}}$) takes the Mendelian sampling term into account, direct genomic heritabilities (Model 2) i.e. 0.33 for BW0, 0.19 for BW23, and 0.22 for BW1314 were lower compared to the pedigree-based heritabilities estimated with the ${\mathbf{A}}$-matrix (Model 1). The lower heritabilities estimated with the ${\mathbf{G}}$-matrix could be due to incomplete LD between SNPs on the 50 K SNP chip and/or very different sample sizes used for the estimations. An explanation for the overestimated pedigree-based heritabilities could be the occurrence of confounding between environmental effects and pedigree relationships [42]. Direct genetic correlations between the three BW traits estimated from genomic relationships (0.51 between BW0 and BW23, 0.33 between BW0 and BW1314, 0.47 between BW23 and BW1314) were slightly higher than those based on pedigree information.

**Table 2:** Genetic parameters for body weight recorded at different ages based on pedigree and genomic relationship matrices

Relationship matrix	Trait	Heritability			Genetic correlation for direct effects	
		Direct	Maternal	Total	BW23	BW1314
Pedigree	BW0	0.46 (0.01)	0.14 (0.01)	0.40 (0.01)	0.46 (0.03)	0.39 (0.03)
	BW23	0.37 (0.01)	0.11 (0.01)	0.23 (0.01)		0.46 (0.04)
	BW1314	0.48 (0.02)	0.13 (0.01)	0.34 (0.01)		
Genomic	BW0	0.33 (0.01)			0.51 (0.05)	0.33 (0.04)
	BW23	0.19 (0.02)				0.47 (0.07)
	BW1314	0.22 (0.02)				

Standard errors in parentheses

BW0: body weight recorded at birth; BW23: body weight recorded at 2 to 3 month of age; BW1314: body weight recorded at 13 to 14 months of age

### Population stratification

**Fig. 1:**
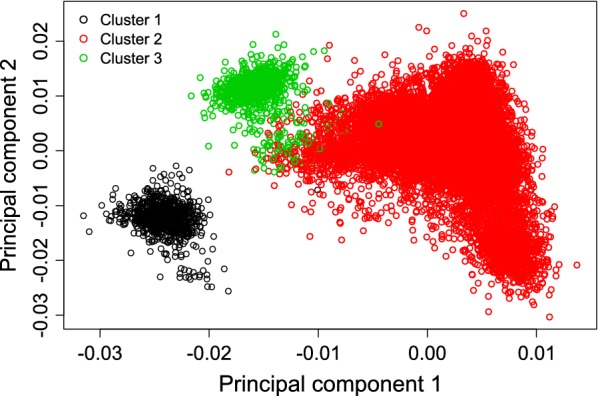
Plot of principal components (PC) 1 and 2 for 15,921 genotyped cows

The k-means clustering approach (using the first 10 PC) created three clusters including 856 cows (cluster 1), 14,305 cows (cluster 2) and 760 cows (cluster 3). Genetic distances between animals based on the two most important PC (the first two PC that contribute to genetic variation) are shown in Fig. 1. Our study included Holstein dairy cattle from only two neighbouring German breeding organizations. When tracing back to the ancestors of the calves and heifers from the three clusters, we found that animals in clusters 1, 2 and 3 were daughters from 2, 890, and 11 sires, respectively. One specific influential sire (Gunnar) in cluster 1 had 855 daughters, whereas another sire (Raik) in cluster 1 had only one daughter in cluster 1 and one daughter in cluster 2 (i.e. the only black dot that overlaps with the red dot in Fig. 1). The 760 calves and heifers in cluster 3 were daughters from 11 different sires. One specific sire (Guarini) had 750 daughters in cluster 3, and the remaining 10 sires only had one daughter each. The maternal grandsire of the nine daughters was Guarini. Sires in cluster 2 originated from various countries, but more than 75% calves and heifers had German and Dutch sires. The remaining 25% females were daughters of sires from 12 other countries. The average number of daughters per sire in cluster 2 was quite small (on average only 16.09). In contrast, the calves and heifers allocated to clusters 1 and 2 were mainly daughters from only two German sires. Consequently, as expected from the pedigree structure, genetic distances between animals within clusters 1 and 2 were short. Hence, the stratification that was observed in the genotyped calves and heifers was mainly due to the size and structure of the half-sib groups. The effect of breeding organization (geographical location) on population stratification was of minor importance, because genotyped animals in all clusters represented both breeding organisations quite equally.

PC1 and PC2 only explained 1.53 and 1.13% of the total genetic variation, respectively. Consequently, we observed several overlaps between the three clusters, especially for animals allocated to clusters 2 and 3. In other studies, population stratification occurred when various breeds were pooled in the same GWAS [43], or because of obvious differences in breeding and selection strategies [44]. In addition, family structure, especially in large families with many closely related paternal half-sibs, generated false positive SNP effects. In this regard, in a preliminary GWAS without considering any polygenic effects, we detected a large number of more than 2000 significant SNPs (after Bonferroni correction), and the inflation factor was equal to 6.04.

### GWAS for body weights

#### Direct genetic effects

The number of significant SNPs that contributed to direct genetic effects for the three BW traits (results from Model 3) are listed in Table 3. Evaluation criteria for all similarity matrices are provided for BW0 only. The general trend in terms of number of significant SNPs and inflation factors for BW23 and BW1314 was in agreement with corresponding similarity matrices for BW0. Inflation factors were largest when using LOCO for the construction of the genetic similarity matrix. This was the case for both types of dependent variables, i.e. phenotypes (inflation factor = 2.22) and dDRP (inflation factor = 2.19). The number of significant SNPs and inflation factors decreased slightly when SNPs on BTA1 to BTA11 were partitioned into two segments (LOCO_SEG40). A further decrease in inflation factor was observed when the number of segments (LOCO_SEG441 and LOCO_SEG864) increased, associated with a reduction of significant SNPs. LOCO plus the first 20 PC of the overall ${\mathbf{G}}$-matrix as covariates identified a quite fairly large number of 73 significant SNPs, and contributed to large inflation factors (1.90 for phenotypes and 1.87 for dDRP). The inclusion of 20 PC of chromosome-wide ${\mathbf{G}}$-matrices as fixed regressions in the model (LOCO + CHR_PC20) decreased the number of significant SNPs, and the inflation factor was close to 1. Inflation factors and number of detected significant SNPs were substantially larger for ${\mathbf{A}}$ (phenotype: 1213 SNPs according to FDR, λ = 1.92) compared to ${\mathbf{G}}$ (phenotype: 7 SNPs according to FDR, λ = 0.96). Such obvious differences were not expected, because ${\mathbf{G}}$ is the realized relationship matrix and ${\mathbf{A}}$ is the expected relationship matrix. Models with ${\mathbf{G}}$ and LOCO + CHR_PC produced inflation factors that were equal to 1.0 or slightly lower than 1.0. Generally, a near identical number of significant SNPs was found in the ${\mathbf{G}}$-matrix scenario and LOCO + CHR_PC scenarios. The number of significant SNPs was larger for BW0 than for BW1314 or BW23. Pruning the SNPs according to low LD decreased inflation factors slightly. This was the case for all three BW traits, regardless of whether phenotypes or dDRP were used as dependent variables. For example, when the phenotype of BW0 was the dependent variable, inflation factors decreased from 2.22 (LOCO) to 1.97 (LOCO_prune). The decrease in inflation factor was even smaller for BW23 and BW1314, which indicated that high LD between SNPs was not the main reason for the large number of false positive SNPs in our dataset.

**Table 3:** Number of significant SNPs influencing direct genetic effects and inflation factor for body weight recorded at different ages

Trait	Dependent variable	Polygenic effect	FDR^a^	Bonferroni^b^	λ
BW0	Phen	${\mathbf{A}}$	1213	64	1.92
		${\mathbf{G}}$	7	3	0.96
		LOCO	2268	125	2.22
		LOCO_pruned	394	43	1.97
		LOCO_SEG40	1811	103	2.06
		LOCO_SEG441	80	17	1.29
		LOCO_SEG864	41	12	1.18
		LOCO + PC20	1132	73	1.90
		LOCO + CHR_PC20	16	7	0.93
	dDRP	${\mathbf{A}}$	1463	104	1.96
		${\mathbf{G}}$	15	6	0.96
		LOCO	2212	163	2.19
		LOCO_pruned	414	54	1.91
		LOCO_SEG40	1802	143	2.07
		LOCO_SEG441	103	23	1.28
		LOCO_SEG864	57	15	1.17
		LOCO + PC20	1180	108	1.87
		LOCO + CHR_PC20	13	8	0.90
BW23	Phen	${\mathbf{A}}$	11	0	1.17
		${\mathbf{G}}$	0	0	0.99
		LOCO + CHR_PC20	0	0	0.66
		LOCO + CHR_PC10	0	0	0.93
	dDRP	${\mathbf{A}}$	22	5	1.33
		${\mathbf{G}}$	3	2	1.00
		LOCO + CHR_PC20	0	0	0.70
		LOCO + CHR_PC10	1	1	0.96
BW1314	Phen	${\mathbf{A}}$	47	14	1.46
		${\mathbf{G}}$	5	3	0.97
		LOCO + CHR_PC20	2	2	0.72
		LOCO + CHR_PC10	7	4	0.98
	dDRP	${\mathbf{A}}$	50	17	1.45
		${\mathbf{G}}$	7	6	0.97
		LOCO + CHR_PC20	3	3	0.72
		LOCO + CHR_PC10	12	6	0.97

FDR: false discovery rate; Bonferroni: Bonferroni correction; λ: inflation factor; BW0: body weight recorded at birth; BW23: body weight recorded at 2 to 3 month of age; BW1314: body weight recorded at 13 to 14 months of age; dDRP: de-regressed proofs for the direct genetic effect; mDRP: de-regressed proofs for the maternal genetic effect; LOCO_pruned: LOCO based on pruned SNPs

^a^Number of significant SNPs according to false discovery rate

^b^Number of significant SNPs according to Bonferroni-correction

Based on our results, it is imperative to correct for population stratification in the German Holstein population via ${\mathbf{G}}$ or ${\mathbf{G}}$-similarities (i.e. the LOCO_CHR_PC-scenarios). GWAS that include multiple breeds and ignore population structure, increased spurious LD, which led to an inflation of false positive signals [43, 45, 46]. Therefore, PC and genetic relationships [15] were included in the GWAS to prevent spurious associations. Yang et al. [34] compared linear mixed models by including or not candidate markers and recommended exclusion of candidate markers from the ${\mathbf{G}}$-matrix because this improved statistical power. However, for the German Holstein population with many closely related animals, LOCO overestimated SNP effects, which indicated that the ${\mathbf{G}}$-matrix from LOCO cannot capture all of the family relatedness. Correlations between the off-diagonal elements from the “full” ${\mathbf{G}}$-matrix and the LOCO ${\mathbf{G}}$-matrix ranged from 0.98 to 1.0, but the LOCO ${\mathbf{G}}$-matrix slightly underestimated the genomic relationships between animals. This underestimation was identified because the regression coefficients were always smaller than 1.0 when regressing relationships from the “full ${\mathbf{G}}$” on relationships from the “LOCO-${\mathbf{G}}$”.

Most of the significant SNPs for direct genetic effects on the three BW traits were located on BTA5 (Table 4). Manhattan and Q–Q plots for direct genetic effects for the three BW traits based on different similarity matrices are presented in Additional file 1. For dDRP of BW23, only two SNPs on BTA5 were significant. Both SNPs were detected using the ${\mathbf{G}}$-matrix. SNP *Hapmap60480*-*ss46526970* was also significant when applying LOCO + CHR_PC. Only two SNPs (*Hapmap60480*-*ss46526970* and *Hapmap57466*-*rs29018274*) on BTA5 significantly contributed to the three BW traits. SNP *Hapmap60480*-*ss46526970* was significant, regardless of the approach applied. However, SNP *Hapmap57466*-*rs29018274* was significant only when the ${\mathbf{G}}$-matrix was considered. The pleiotropic SNP (*Hapmap60480*-*ss46526970*) on BTA5, and the significant SNP on BTA18 (*ARS*-*BFGL*-*NGS*-*109285*), also contributed significantly to BW changes in genotyped Holstein dairy cows in the US [47]. On BTA18, SNP *ARS*-*BFGL*-*NGS*-*109285* was significantly associated with body shape, body size, dystocia, longevity, lifetime economic merit [48], and calving difficulty [15]. The four significant SNPs, i.e. *ARS*-*BFGL*-*NGS*-*39379* for BW0 and BW1314, *ARS*-*BFGL*-*NGS*-*5139* for BW0, *ARS*-*BFGL*-*NGS*-*107035* for BW0, and *ARS*-*BFGL*-*NGS*-*109317* for BW0, had a significant impact on BW [49], live weight [50], carcass retail beef yield [43], and hot carcass weight [51] in beef and crossbred beef cattle.

**Table 4:** Significant SNPs according to Bonferroni correction for direct genetic effects on body weight recorded at different ages

SNP	Chr	Position	Ref allele	Effect	BW0		BW23		BW1314	
					Phen	dDRP	Phen	dDRP	Phen	dDRP
*INRA*-*658*	3	29627982	A	−						X^c^
*BTB*-*01695573*	4	10794285	C	+	X^b^	X^b^				
*ARS*-*BFGL*-*NGS*-*3933*	5	105695909	G	−		X^a^				
*Hapmap47397*-*BTA*-*74925*	5	105744830	A	−	X^ab^	X^ab^				X^ac^
*Hapmap60480*-*ss46526970*	5	105870613	C	−	X^ab^	X^ab^		X^ac^	X^ac^	X^ac^
*ARS*-*BFGL*-*NGS*-*39379*	5	106269362	G	−	X^ab^	X^ab^			X^c^	X^ac^
*ARS*-*BFGL*-*NGS*-*10732*	5	106780606	G	−					X^ac^	X^ac^
*Hapmap57466*-*rs29018274*	5	107362671	A	+		X^a^		X^a^		X^a^
*ARS*-*BFGL*-*NGS*-*5139*	7	92474466	A	+		X^b^				
*ARS*-*BFGL*-*NGS*-*107035*	7	93007435	A	+	X^b^	X^ab^				
*ARS*-*BFGL*-*NGS*-*109285*	18	57589121	A	+		X^b^			X^ac^	X^ac^
*ARS*-*BFGL*-*NGS*-*109317*	29	49906123	A	+	X^b^					
*ARS*-*BFGL*-*NGS*-*40378*	29	50296573	A	+	X^b^	X^b^				

BW0: body weight recorded at birth; BW23: body weight recorded at 2 to 3 month of age; BW1314: body weight recorded at 13 to 14 months of age

The indicated significant SNPs are from runs that consider the following similarity matrices: ^a^the genomic relationship matrix ${\mathbf{G}}$, ^b^LOCO + CHR_PC20 and ^c^LOCO + CHR_PC10

#### Maternal genetic effects

**Fig. 2:**
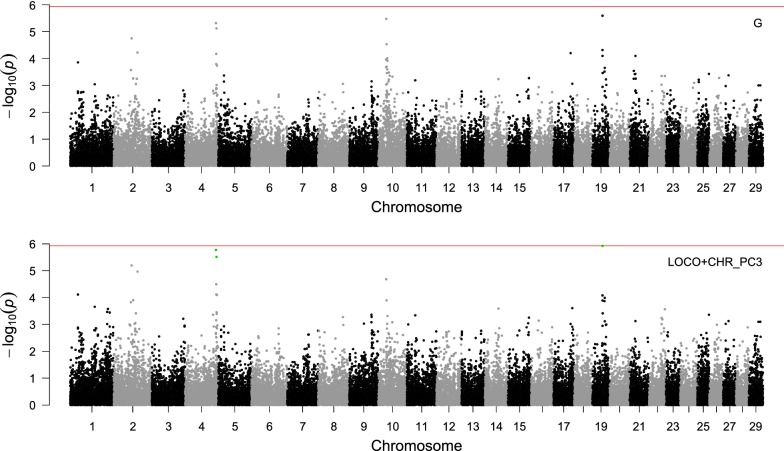
Manhattan plot from GWAS for maternal genetic effects on birth weight. ${\mathbf{G}}$: Genomic relationship matrix; LOCO + CHR_PC3: leave-one-chromosome-out plus 3 principal components based on the chromosomal genomic relationship matrix. The red line is the significance threshold line for the Bonferroni correction of 5%, and the green dots represent significant SNPs according to a false discovery rate of 5%

For maternal genetic effects, only three significant SNPs according to the FDR threshold were identified when using LOCO plus chromosome-wide PC (Table 5). Two SNPs located on BTA4 and one SNP on BTA19 influenced BW0 significantly (Table 6). Regarding maternal genetic effects at later age points for BW23 and BW1314, no significant SNP was detected. The Manhattan plots for maternal genetic effects on BW0 are in Fig. 2. In a study conducted in crossbred beef cattle [51], the significant SNP *ARS*-*BFGL*-*NGS*-*61198* on BTA4 explained 2.67% of the phenotypic variation for lean rate. The significant SNP *Hapmap53086*-*rs29025958* on BTA19 was identified as a marker for a QTL that controls fat percentage [52]. According to the infinitesimal model for maternal effects on calving performance [15], many genes with small effects influenced the maternal effect on BW. In this regard, the most significant SNP on BTA 19 explained only 0.14% of the mDRP variance for BW0.

**Table 5:** Number of significant SNPs influencing maternal genetic effects and inflation factor for body weight recorded at different ages

Trait	Dependent variable	Polygenic effect	FDR^a^	Bonferroni^b^	λ
BW0	mDRP	${\mathbf{A}}$	26	6	1.12
		${\mathbf{G}}$	0	0	0.99
		LOCO + CHR_PC20	0	0	0.64
		LOCO + CHR_PC3	3	0	1.00
BW23	mDRP	${\mathbf{A}}$	0	0	1.04
		${\mathbf{G}}$	0	0	0.99
		LOCO + CHR_PC20	0	0	0.59
		LOCO + CHR_PC3	0	0	0.91
BW1314	mDRP	${\mathbf{A}}$	0	0	1.08
		${\mathbf{G}}$	0	0	1.00
		LOCO + CHR_PC20	0	0	0.56
		LOCO + CHR_PC3	0	0	0.91

FDR: false discovery rate; Bonferroni: Bonferroni correction; λ: inflation factor; BW0: body weight recorded at birth; BW23: body weight recorded at 2 to 3 months of age; BW1314: body weight recorded at 13 to 14 months of age; dDRP: de-regressed proofs for the direct genetic effect; mDRP: de-regressed proofs for the maternal genetic effect

^a^Number of significant SNPs according to false discovery rate

^b^Number of significant SNPs according to Bonferroni-correction

**Table 6:** Significant SNPs according to false discover rate for maternal-genetic effects on body weight recorded at different ages

SNP	Chr	Position	Ref. allele	Effect	BW0 mDRP	BW23 mDRP	BW1314 mDRP
*ARS*-*BFGL*-*NGS*-*61198*	4	112474006	A	+	X		
*ARS*-*BFGL*-*NGS*-*107181*	4	114464406	A	+	X		
*Hapmap53086*-*rs29025958*	19	37626478	G	+	X		

BW0: body weight recorded at birth; BW23: body weight recorded at 2 to 3 month of age; BW1314: body weight recorded at 13 to 14 months of age

The indicated significant SNPs are from the run that consider the similarity matrix: LOCO + CHR_PC3

**Fig. 3:**
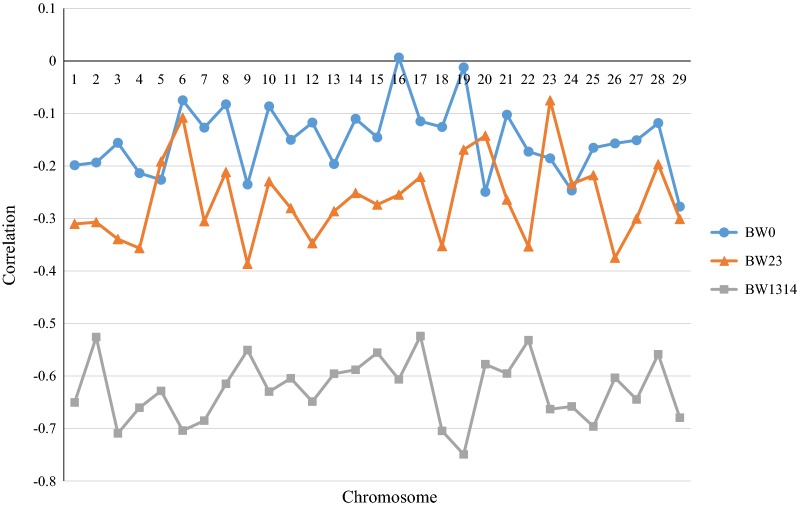
Correlations between direct genetic and maternal genetic marker effects for body weight recorded at birth (BW0), at 2 to 3 months of age (BW23) and at 13 to 14 months of age (BW1314)

Correlations between SNP effects (using DRP and the ${\mathbf{G}}$-matrix in Model 3) for direct genetic and maternal genetic effects were − 0.15 for BW0, − 0.27 for BW23, and − 0.62 for BW1314. Antagonistic correlations between SNP effects for direct genetic and maternal genetic effects for each chromosome were identified for all three BW traits, except for BW0 (0.01) on BTA16 (Fig. 3). In agreement with correlations that take the SNPs on all the chromosomes into account, and in agreement with pedigree-based correlations, antagonistic relationships between direct genetic and maternal genetic effects were most obvious for BW1314. If we focus on the functional region on BTA5 (i.e. between 105,445,909 and 107,612,671 bp), the direct-maternal correlations based on the effects of 46 SNPs were equal to − 0.04 for BW0, 0.06 for BW23, and − 0.87 for BW1314.

### Genomic heritability for each chromosome

**Fig. 4:**
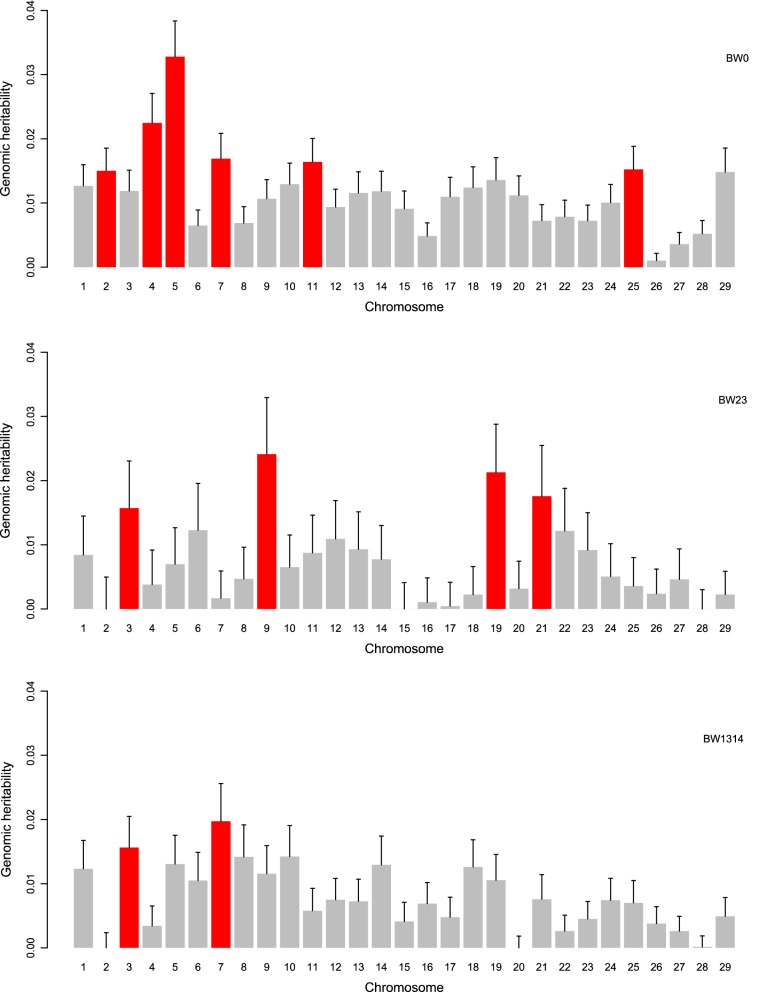
Chromosomal genomic heritabilities for direct genetic effects of body weights recorded at birth (BW0), at 2 to 3 months of age (BW23) and at 13 to 14 months of age (BW1314). The red bars represent chromosomes with genomic heritabilities higher than 0.015

Genomic heritabilities for the three BW traits across the 29 bovine autosomes (results from Model 4) are in Fig. 4. For BW0, genomic heritability was highest (0.03) when the SNPs on BTA5 were considered and decreased to 0.001 when those on BTA26 were considered. BTA5 and BTA26 explained 9.92 and 0.31% of the total genomic variance for BW0, respectively. Genomic heritabilities higher than 0.015 were estimated for BTA2, 4, 5, 7, 11, and 25. When comparing chromosomal genomic variances with GWAS results for BW0 (see Additional file 1a and c), the proportion of explained genomic variance increased as the number of significant SNPs per chromosome increased. For BW23, genomic heritabilities were lower than 0.001 for BTA2, 15, and 28, higher than 0.015 for BTA3, 9, 19 and 21, but significant SNPs were detected only on BTA5 (see Additional file 1e). For BW1314, the highest genomic heritability (0.02) was found for BTA7, but significant SNPs were detected on BTA3, 5, 8, 16, and 18 (see Additional file 1g), for which genomic heritabilities were higher than 0.012 for BTA3, 5, 8, and 18 and only 0.007 for BTA16.

Heterogeneous chromosomal contributions were also reported for BW in Korean beef cattle [53]. Consistent with the latter study, we found variations in chromosome-wise BW variances for the same chromosomes at different ages. Hence, such changes in genomic variances indicate that the genetic mechanisms underlying BW differ with age, i.e. that different genes are “switched on or off” during the growth period. We have identified some chromosomes that explain more than 0.015% of the total genomic variance, although no significant SNP was detected (BTA9 for BW23 and BTA7 for BW1314), which indicates polygenic contribution to BW on these chromosomes.

In contrast to [54], we found weak negative covariances between chromosome-wise genomic effects in our data, because the proportions of the sum of chromosome-wise variances to total genomic variances reached 100.81% for BW0, 106.56% for BW23, and 101.41% for BW1314. Linear associations between chromosome length and chromosomal genomic variances were weak for BW0 and BW1314, with R^2^ values of 0.20 and 0.21, respectively, and null for BW23 (R^2^ = 0.02). Weak associations between chromosome length and chromosomal genomic variances indicate that the QTL for BW are not evenly distributed across the genome [54].

### Gene annotation

#### Direct genetic effect

The identified potential candidate genes that significantly influence direct genetic effects on BW are in Additional file 2. These candidate genes are located on 12 chromosomes: BTA3, 4, 5, 7, 8, 11, 13, 18, 19, 23, 25 and 29, which BTA5 and BTA18 carrying more than ten. Overall, for the three BW traits, 76 potential candidate genes had adjusted *p* values lower than 0.05 (according to FDR), with 51 significant genes for BW0, 12 for BW23, and 38 for BW1314; these figures reflect the smaller number of significant SNPs detected in the GWAS for BW at later ages. Six genes contributed significantly to the three BW traits and 12 more contributed to both BW0 and BW1314, but only one more gene, i.e. *fast skeletal muscle troponin T* (*TNNT3*) had a significant effect on both BW0 and BW23. Low to moderate genetic correlations between BW traits at different age points, but with some overlapping between significant genes, could indicate pleiotropic effects of the candidate genes.

Some of the potential candidate genes on BTA18 for BW traits are known to be involved in calving performance and conformation traits. For example, Abo-Ismail et al. [16] reported that *cytosolic thiouridylase subunit 1* (*CTU1*) and *ENSBTAG00000037537* are highly associated with body conformation traits and *kallikrein related peptidase 4* (*KLK4*), *CTU1* and *ENSBTAG00000004608* contributes to calving ease. Purfield et al. [15] showed that *CTU1* and *ENSBTAG00000037537* contain one and two significant missense variants, respectively, that are associated with calving difficulty in a mixed bull population including Holstein–Friesian, Charolais and Limousin. Since the above-mentioned six genes also influence birth weight, the calving difficulties in these breeds are mainly due to increased BW of the newborn [55].

**Fig. 5:**
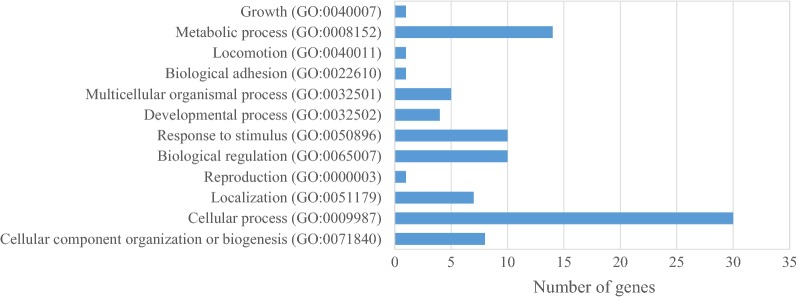
Biological processes for direct genetic effects on body weight at different ages

Our analyses revealed that the identified potential candidate genes were involved in 12 biological processes (Fig. 5): cellular processes (30 genes), metabolic mechanisms (14 genes), biological regulations and responses to stimuli (10 genes), growth (one gene), and body developmental processes (four genes). The latter four genes were *fibroblast growth factors 6* (*FGF6*) and *fibroblast growth factors 23* (*FGF23*), *fast skeletal muscle troponin T* (*TNNT3*), and o*steomodulin* (*OMD*). *FGF6* and *FGF23* belong to the fibroblast growth factor family, which plays an important role in a variety of biological processes, including angiogenesis, morphogenesis, tissue regeneration, and oncogenesis [40]. Another significant gene, i.e. *cathepsin D* (*CTSD*) is involved in the activation and degradation of polypeptide hormones and growth factors [56]. *TNNT3* produces troponin T protein in the mammalian fast skeletal muscle, with causal effects on Ca^2+^ muscle contractions [57]. *OMD* regulates the diameter and shape of collagen fibrils, which suggest an effect on bone formation [58].

#### Maternal genetic effect

Four potential candidate genes on BTA19, i.e. *solute carrier family 35 member B1* (SLC35B1), *speckle*-*type POZ protein* (*SPOP*), *neurexophilin 3* (*NXPH3*), and *nerve growth factor receptor* (*NGFR*), were significantly associated with birth weight (see Additional file 3), although only one significant SNP was detected on BTA19. The biological functions of *SLC35B1* and *NXPH3* remain unknown. *SPOP* is an important regulator of luminal epithelial cell proliferation [59] and is associated with various cancers. *NGFR* affects cell growth and survival [60]. None of these four genes overlapped with the candidate genes identified for direct genetic effects.

## Conclusions

Ignoring the population structure of Holstein–Friesian in the GWAS increased the number of false positive SNPs. Population structure was corrected properly when using ${\mathbf{G}}$ and LOCO plus chromosome-wide PC in the statistical models for the GWAS. The number of significant SNPs increased when DRP instead of phenotypes were used as dependent variables. Two SNPs on BTA5 influenced direct genetic effects significantly for BW at the three ages measured. Chromosomes with a larger number of significant SNPs had higher direct chromosomal heritabilities. Gene annotation analysis identified 76 potential candidate genes that are involved in 12 biological processes, which indicates that weight development is a very complex biological process. Regarding birth weight, only a limited number of significant SNPs and candidate genes were identified for the maternal genetic effects, which suggests an infinitesimal model for these effects. Antagonistic associations between direct genetic and maternal genetic effects were observed both when SNPs on all bovine chromosomes or on single chromosomes were considered, and for potential functional regions on BTA5.

## Additional files

**Additional file 1.** Manhattan plots and Q–Q plots from GWAS for birth weight phenotypes (a and b), for birth weight de-regressed proofs (c and d), for BW23 phenotypes and de-regressed proofs (e and f), and for BW1314 phenotypes and de-regressed proofs (g and h). Description: ${\mathbf{A}}$ is the pedigree-based relationship matrix; ${\mathbf{G}}$ is the genomic relationship matrix; LOCO is leave-one-chromosome-out (LOCO); LOCO_SEG864 is leave one segment out; LOCO + PC20 is LOCO plus 20 principal components; LOCO + CHR_PC20 is LOCO plus 20 principal components based on the chromosomal genomic relationship matrix. The red line is the significance threshold line for the Bonferroni correction of 5%, and the green dots represent significant SNPs according to ae false discovery rate of 5%.

**Additional file 2.** Potential candidate genes for direct genetic effects on body weight recorded at birth (BW0), at 2 to 3 months of age (BW23) and at 13 to 14 months of age (BW1314).

**Additional file 3.** Potential candidate genes for maternal genetic effects on body weights recorded at birth (BW0), at 2 to 3 months of age (BW23) and at 13 to 14 months of age (BW1314).

## References

[1] DairyNZ. https://www.dairynz.co.nz/animal/animal-evaluation/interpreting-the-info/breeding-values/. Accessed 13 Jul 2017.

[2] Australian Dairy Herd Improvement Scheme. https://www.adhis.com.au/. Accessed 13 Jul 2017.

[3] Veerkamp RF, Oldenbroek JK, van der Gaast HJ, van der Werf JH (2000). Genetic correlation between days until start of luteal activity and milk yield, energy balance, and live weights. J Dairy Sci.

[4] Yin T, König S (2018). Genetic parameters for body weight from birth to calving and associations between weights with test-day, health, and female fertility traits. J Dairy Sci.

[5] Berry DP, Buckley F, Dillon P, Evans RD, Rath M, Veerkamp RF (2003). Genetic relationships among body condition score, body weight, milk yield, and fertility in dairy cows. J Dairy Sci.

[6] Britt JS, Thomas RC, Speer NC, Hall MB (2003). Efficiency of converting nutrient dry matter to milk in Holstein herds. J Dairy Sci.

[7] Coleman J, Berry DP, Pierce KM, Brennan A, Horan B (2010). Dry matter intake and feed efficiency profiles of 3 genotypes of Holstein-Friesian within pasture-based systems of milk production. J Dairy Sci.

[8] Pryce JE, Gonzalez-Recio O, Nieuwhof G, Wales WJ, Coffey MP, Hayes BJ (2015). Hot topic: definition and implementation of a breeding value for feed efficiency in dairy cows. J Dairy Sci.

[9] Johanson JM, Berger PJ, Tsuruta S, Misztal I (2011). A Bayesian threshold-linear model evaluation of perinatal mortality, dystocia, birth weight, and gestation length in a Holstein herd. J Dairy Sci.

[10] Pryce JE, Arias J, Bowman PJ, Davis SR, Macdonald KA, Waghorn GC (2012). Accuracy of genomic predictions of residual feed intake and 250-day body weight in growing heifers using 625,000 single nucleotide polymorphism markers. J Dairy Sci.

[11] Cole JB, Waurich B, Wensch-Dorendorf M, Bickhart DM, Swalve HH (2014). A genome-wide association study of calf birth weight in Holstein cattle using single nucleotide polymorphisms and phenotypes predicted from auxiliary traits. J Dairy Sci.

[12] Zhang X, Chu Q, Guo G, Dong G, Li X, Zhang Q (2017). Genome-wide association studies identified multiple genetic loci for body size at four growth stages in Chinese Holstein cattle. PLoS ONE.

[13] Schaeffer LR. Maternal genetic models. http://www.aps.uoguelph.ca/%7Elrs/ABModels/NOTES/matern.pdf. Accessed 7 Mar 2018.

[14] Olsen HG, Hayes BJ, Kent MP, Nome T, Svendsen M, Lien S (2010). A genome wide association study for QTL affecting direct and maternal effects of stillbirth and dystocia in cattle. Anim Genet.

[15] Purfield DC, Bradley DG, Evans RD, Kearney FJ, Berry DP (2015). Genome-wide association study for calving performance using high-density genotypes in dairy and beef cattle. Genet Sel Evol.

[16] Abo-Ismail MK, Brito LF, Miller SP, Sargolzaei M, Grossi DA, Moore SS (2017). Genome-wide association studies and genomic prediction of breeding values for calving performance and body conformation traits in Holstein cattle. Genet Sel Evol.

[17] Goddard ME, Hayes BJ (2009). Mapping genes for complex traits in domestic animals and their use in breeding programmes. Nat Rev Genet.

[18] Coffey MP, Hickey J, Brotherstone S (2006). Genetic aspects of growth of Holstein-Friesian dairy cows from birth to maturity. J Dairy Sci.

[19] Naderi S, Yin T, König S (2016). Random forest estimation of genomic breeding values for disease susceptibility over different disease incidences and genomic architectures in simulated cow calibration groups. J Dairy Sci.

[20] Price AL, Zaitlen NA, Reich D, Patterson N (2010). New approaches to population stratification in genome-wide association studies. Nat Rev Genet.

[21] MacLeod IM, Hayes BJ, Savin KW, Chamberlain AJ, McPartlan HC, Goddard ME (2010). Power of a genome scan to detect and locate quantitative trait loci in cattle using dense single nucleotide polymorphisms. J Anim Breed Genet.

[22] Yang J, Weedon MN, Purcell S, Lettre G, Estrada K, Willer CJ (2011). Genomic inflation factors under polygenic inheritance. Eur J Hum Genet.

[23] Fox J, Weisberg S. An R Companion to applied regression. 2nd ed. London: SAGE Publications Ltd; 2011. Accessed 17 Jul 2017.

[24] Segelke D, Chen J, Liu Z, Reinhardt F, Thaller G, Reents R (2012). Reliability of genomic prediction for German Holsteins using imputed genotypes from low-density chips. J Dairy Sci.

[25] Aulchenko YS, Ripke S, Isaacs A, van Duijn CM (2007). GenABEL: an R library for genome-wide association analysis. Bioinformatics.

[26] Purcell S, Neale B, Todd-Brown K, Thomas L, Ferreira MAR, Bender D (2007). PLINK: a tool set for whole-genome association and population-based linkage analyses. Am J Hum Genet.

[27] Pausch H, Flisikowski K, Jung S, Emmerling R, Edel C, Götz KU (2011). Genome-wide association study identifies two major loci affecting calving ease and growth-related traits in cattle. Genetics.

[28] Yang J, Benyamin B, McEvoy BP, Gordon S, Henders AK, Nyholt DR (2010). Common SNPs explain a large proportion of the heritability for human height. Nat Genet.

[29] Yang J, Lee SH, Goddard ME, Visscher PM (2011). GCTA: a tool for genome-wide complex trait analysis. Am J Hum Genet.

[30] Misztal I, Tsuruta S, Strabel T, Auvray B, Druet T, Lee DH. BLUPF90 and related programs (BGF90). In: Proceedings of the 7th world congress on genetics applied to livestock production: 19–23 August 2002. Montpellier; 2002.

[31] Garrick DJ, Taylor JF, Fernando RL (2009). Deregressing estimated breeding values and weighting information for genomic regression analyses. Genet Sel Evol.

[32] Benjamini Y, Hochberg Y (1995). Controlling the false discovery rate: a practical and powerful approach to multiple testing. J R Stat Soc Ser B.

[33] Listgarten J, Lippert C, Kadie CM, Davidson RI, Eskin E, Heckerman D (2012). Improved linear mixed models for genome-wide association studies. Nat Methods.

[34] Yang J, Zaitlen NA, Goddard ME, Visscher PM, Price AL (2014). Advantages and pitfalls in the application of mixed-model association methods. Nat Genet.

[35] Power RA, Parkhill J, Oliveira T (2017). Microbial genome-wide association studies: lessons from human GWAS. Nat Rev Genet.

[36] Ensembl-BioMart. http://www.ensembl.org/biomart/martview/. Accessed 19 Apr 2017.

[37] Ashburner M, Ball CA, Blake JA, Botstein D, Butler H, Cherry JM (2000). Gene ontology: tool for the unification of biology. The Gene Ontology Consortium. Nat Genet.

[38] The Gene Ontology Consortium (2017). Expansion of the gene ontology knowledgebase and resources. Nucleic Acids Res.

[39] de Leeuw CA, Mooij JM, Heskes T, Posthuma D (2015). MAGMA: generalized gene-set analysis of GWAS data. PLoS Comput Biol.

[40] Coulier F, Batoz M, Marics I, de Lapeyriere O, Birnbaum D (1991). Putative structure of the FGF6 gene product and role of the signal peptide. Oncogene.

[41] Brotherstone S, Coffey MP, Banos G (2007). Genetic parameters of growth in dairy cattle and associations between growth and health traits. J Dairy Sci.

[42] Lee SH, Goddard ME, Visscher PM, van der Werf JH (2010). Using the realized relationship matrix to disentangle confounding factors for the estimation of genetic variance components of complex traits. Genet Sel Evol.

[43] Bolormaa S, Pryce JE, Kemper K, Savin K, Hayes BJ, Barendse W (2013). Accuracy of prediction of genomic breeding values for residual feed intake and carcass and meat quality traits in *Bos taurus*, *Bos indicus*, and composite beef cattle. J Anim Sci.

[44] Bergfelder-Drüing S, Grosse-Brinkhaus C, Lind B, Erbe M, Schellander K, Simianer H (2015). A genome-wide association study in large white and landrace pig populations for number piglets born alive. PLoS ONE.

[45] Price AL, Patterson NJ, Plenge RM, Weinblatt ME, Shadick NA, Reich D (2006). Principal components analysis corrects for stratification in genome-wide association studies. Nat Genet.

[46] Pausch H, Jung S, Edel C, Emmerling R, Krogmeier D, Götz K-U (2012). Genome-wide association study uncovers four QTL predisposing to supernumerary teats in cattle. Anim Genet.

[47] Lu Y. Quantitative genetic and genomic modeling of feed efficiency in dairy cattle. PhD thesis, Michigan State University. 2016.

[48] Cole JB, Hutchison JL, Null DJ, VanRaden PM, Liu GE, Schroeder TP, et al. The hunt for a functional mutation affecting conformation and calving traits on chromosome 18 in Holstein cattle. In: Proceedings of the 10th world congress of genetics applied to livestock production: 17–22 August 2014. Vancouver; 2014.

[49] Blackburn HD, Krehbiel B, Ericsson SA, Wilson C, Caetano AR, Paiva SR (2017). A fine structure genetic analysis evaluating ecoregional adaptability of a *Bos taurus* breed (Hereford). PLoS ONE.

[50] Veerkamp RF, Coffey MP, Berry DP, de Haas Y, Strandberg E, Bovenhuis H (2012). Genome-wide associations for feed utilisation complex in primiparous Holstein-Friesian dairy cows from experimental research herds in four European countries. Animal.

[51] Lu D, Sargolzaei M, Kelly M, Vander Voort G, Wang Z, Mandell I (2013). Genome-wide association analyses for carcass quality in crossbred beef cattle. BMC Genet.

[52] Zhang Z, Ober U, Erbe M, Zhang H, Gao N, He J (2014). Improving the accuracy of whole genome prediction for complex traits using the results of genome wide association studies. PLoS ONE.

[53] Ryu J, Lee C (2014). Genomic heritability of bovine growth using a mixed model. Asian Australas J Anim Sci.

[54] Jensen J, Su G, Madsen P (2012). Partitioning additive genetic variance into genomic and remaining polygenic components for complex traits in dairy cattle. BMC Genet.

[55] Gutierrez JP, Goyache F, Fernandez I, Alvarez I, Royo LJ (2007). Genetic relationships among calving ease, calving interval, birth weight, and weaning weight in the Asturiana de los Valles beef cattle breed. J Anim Sci.

[56] Benes P, Vetvicka V, Fusek M (2008). Cathepsin D–many functions of one aspartic protease. Crit Rev Oncol Hematol.

[57] Chaudhuri T, Mukherjea M, Sachdev S, Randall JD, Sarkar S (2005). Role of the fetal and alpha/beta exons in the function of fast skeletal troponin T isoforms: Correlation with altered Ca^2+^ regulation associated with development. J Mol Biol.

[58] Tashima T, Nagatoishi S, Sagara H, Ohnuma SI, Tsumoto K (2015). Osteomodulin regulates diameter and alters shape of collagen fibrils. Biochem Biophys Res Commun.

[59] Geng C, Kaochar S, Li M, Rajapakshe K, Fiskus W, Dong J (2017). SPOP regulates prostate epithelial cell proliferation and promotes ubiquitination and turnover of c-MYC oncoprotein. Oncogene.

[60] Nakamura T, Endo K-I, Kinoshita S (2007). Identification of human oral keratinocyte stem/progenitor cells by neurotrophin receptor p75 and the role of neurotrophin/p75 signaling. Stem Cells.

