# Supplementary material for: Genome-wide associations and detection of potential candidate genes for direct genetic and maternal genetic effects influencing dairy cattle body weight at different ages
Source: Genet Sel Evol. 2019 Feb 6;51:4. doi: 10.1186/s12711-018-0444-4 (PMC6366057; doi:10.1186/s12711-018-0444-4)
Supplement: Supplementary file 1 — Additional file 1. Manhattan plots and Q–Q plots from GWAS for birth weight phenotypes (a and b), for birth weight de-regressed proofs (c and d), for BW23 phenotypes and de-regressed proofs (e and f), and for BW1314 phenotypes and de-regressed proofs (g and h). Description: ${\mathbf{A}}$A is the pedigree-based relationship matrix; ${\mathbf{G}}$G is the genomic relationship matrix; LOCO is leave-one-chromosome-out (LOCO); LOCO_SEG864 is leave one segment out; LOCO + PC20 is LOCO plus 20 principal components; LOCO + CHR_PC20 is LOCO plus 20 principal components based on the chromosomal genomic relationship matrix. The red line is the significance threshold line for the Bonferroni correction of 5%, and the green dots represent significant SNPs according to ae false discovery rate of 5%. [file 12711_2018_444_MOESM1_ESM.docx]

**Additional file 1****.** **Manhattan plots and Q-Q plots from GWAS for birth weight phenotypes (a and b), for birth weight de-regressed proofs (c and d), for BW23 phenotypes and de-regressed proofs (e and f), and for BW1314 phenotypes and de-regressed proofs (g and h). A**: pedigree based relationship matrix; **G**: genomic relationship matrix; LOCO: leave-one-chromosome-out (LOCO); LOCO_SEG864: leave one segment out; LOCO+PC20: LOCO plus 20 principal components; LOCO+CHR_PC20: LOCO plus 20 principal components based on the chromosomal genomic relationship matrix. The red line is the significance threshold line for the Bonferroni correction of 5%, and the green dots represent significant SNP according to the false discovery rate of 5%.

**a**

**b**

**c**

**d**

**e**

**f**

**g**

**h**
