# Supplementary material for: Genome-wide associations and detection of potential candidate genes for direct genetic and maternal genetic effects influencing dairy cattle body weight at different ages
Source: Genet Sel Evol. 2019 Feb 6;51:4. doi: 10.1186/s12711-018-0444-4 (PMC6366057; doi:10.1186/s12711-018-0444-4)
Supplement: Supplementary file 2 — Additional file 2. Potential candidate genes for direct genetic effects on body weight recorded at birth (BW0), at 2 to 3 months of age (BW23) and at 13 to 14 months of age (BW1314). [file 12711_2018_444_MOESM2_ESM.docx]

**Additional file 2. Potential candidate genes for direct genetic effects on body weights recorded at birth (BW0), at age month 2 to 3 (BW23) and at age month 13 to 14 (BW1314).**

| **Gene** | **Chr.** | **Start** | **End** | **BW0** | **BW23** | **BW1314** |
| --- | --- | --- | --- | --- | --- | --- |
| *SYT6* | 3 | 29343182 | 29402185 |  |  | X |
| *HIPK1* | 3 | 29512758 | 29563150 |  |  | X |
| *DCLRE1B* | 3 | 29618297 | 29624169 |  |  | X |
| *AP4B1* | 3 | 29624719 | 29635004 |  |  | X |
| *BCL2L15* | 3 | 29643176 | 29648016 |  |  | X |
| *PTPN22* | 3 | 29666149 | 29722631 |  |  | X |
| *RSBN1* | 3 | 29725839 | 29765019 |  |  | X |
| *ENSBTAG00000045677* | 4 | 107295010 | 107297429 | X |  |  |
| *ENSBTAG00000006655* | 4 | 107308521 | 107309079 | X |  |  |
| *ENSBTAG00000031212* | 4 | 107344454 | 107347462 | X |  |  |
| *NACA* | 5 | 57063071 | 57074070 |  |  | X |
| *PTGES3* | 5 | 57091367 | 57112468 |  |  | X |
| *SYN3* | 5 | 71475847 | 71926718 | X |  |  |
| *KCNA1* | 5 | 105663225 | 105664712 | X | X | X |
| *KCNA6* | 5 | 105762455 | 105764041 | X | X | X |
| *GALNT8* | 5 | 105799704 | 105837839 | X | X | X |
| *NDUFA9* | 5 | 105870530 | 105892347 | X | X | X |
| *AKAP3* | 5 | 105896016 | 105941353 | X | X | X |
| *DYRK4* | 5 | 105946100 | 105985653 | X | X | X |
| *FGF6* | 5 | 106157909 | 106169922 | X |  | X |
| *FGF23* | 5 | 106208179 | 106216757 | X |  | X |
| *TIGAR* | 5 | 106223071 | 106238040 | X |  | X |
| *CCND2* | 5 | 106253907 | 106276819 | X |  | X |
| *TULP3* | 5 | 107343179 | 107373637 |  | X |  |
| *RHNO1* | 5 | 107374654 | 107377720 |  | X |  |
| *FOXM1* | 5 | 107386227 | 107397369 |  | X |  |
| *NRIP2* | 5 | 107418042 | 107424743 |  | X |  |
| *ITFG2* | 5 | 107426529 | 107436938 |  | X |  |
| *MBLAC2* | 7 | 92366907 | 92391409 | X |  |  |
| *POLR3G* | 7 | 92404697 | 92434910 | X |  |  |
| *LYSMD3* | 7 | 92440985 | 92446470 | X |  |  |
| *ZNF484* | 8 | 85224607 | 85232666 |  |  | X |
| *IARS* | 8 | 85268890 | 85350117 |  |  | X |
| *OMD* | 8 | 85477017 | 85479293 |  |  | X |
| *ASPN* | 8 | 85499333 | 85524670 |  |  | X |
| *ECM2* | 8 | 85540501 | 85579683 |  |  | X |
| *PNPLA7* | 11 | 105598761 | 105673200 | X |  |  |
| *CSTF1* | 13 | 60077491 | 60091145 | X |  |  |
| *COMMD7* | 13 | 62647738 | 62675887 | X |  |  |
| *KLK14* | 18 | 57495324 | 57499297 | X |  | X |
| *CTU1* | 18 | 57517497 | 57524958 | X |  | X |
| *ENSBTAG00000004608* | 18 | 57574117 | 57583637 | X |  | X |
| *ENSBTAG00000037537* | 18 | 57588055 | 57594260 | X |  | X |
| *ENSBTAG00000030440* | 18 | 57612011 | 57616992 | X |  | X |
| *ENSBTAG00000047301* | 18 | 57618759 | 57622795 | X |  | X |
| *ENSBTAG00000037710* | 18 | 57630986 | 57632950 | X |  | X |
| *ENSBTAG00000037699* | 18 | 57640179 | 57681883 | X |  | X |
| *VSTM1* | 18 | 62145373 | 62165477 |  |  | X |
| *ENSBTAG00000023349* | 18 | 62194105 | 62195025 |  |  | X |
| *NLRP9* | 18 | 62219311 | 62245235 |  |  | X |
| *BOSTAUV1R424* | 18 | 62259970 | 62260863 |  |  | X |
| *EPN1* | 18 | 62316246 | 62324320 |  |  | X |
| *MIF4GD* | 19 | 56855652 | 56859258 | X |  |  |
| *MRPS7* | 19 | 56859683 | 56862920 | X |  |  |
| *GGA3* | 19 | 56863207 | 56877029 | X |  |  |
| *NUP85* | 19 | 56877689 | 56905608 | X |  |  |
| *HN1* | 19 | 56938745 | 56953081 | X |  |  |
| *NT5C* | 19 | 56955613 | 56957740 | X |  |  |
| *ARMC7* | 19 | 56957747 | 56972759 | X |  |  |
| *RIPOR2* | 23 | 32681207 | 32763526 |  |  | X |
| *TMEM184A* | 25 | 41946139 | 41956711 | X |  |  |
| *MAFK* | 25 | 41960757 | 41961974 | X |  |  |
| *INTS1* | 25 | 41983696 | 42007511 | X |  |  |
| *GPER1* | 25 | 42259657 | 42260709 | X |  |  |
| *CYP2W1* | 25 | 42306360 | 42310487 | X |  |  |
| *COX19* | 25 | 42316375 | 42321964 | X |  |  |
| *ADAP1* | 25 | 42330026 | 42362918 | X |  |  |
| *TRPM5* | 29 | 49817287 | 49835313 | X |  |  |
| *CD81* | 29 | 49842969 | 49848639 | X |  |  |
| *TSPAN32* | 29 | 49879043 | 49926003 | X |  |  |
| *TNNT3* | 29 | 50218484 | 50233948 | X | X |  |
| *LSP1* | 29 | 50238211 | 50276209 | X |  |  |
| *TNNI2* | 29 | 50285049 | 50287648 | X |  |  |
| *SYT8* | 29 | 50289094 | 50293879 | X |  |  |
| *CTSD* | 29 | 50352064 | 50361497 | X |  |  |
| *CRLF2* | 29 | 50372337 | 50386295 | X |  |  |
