# Supplementary material for: Genome-wide associations and detection of potential candidate genes for direct genetic and maternal genetic effects influencing dairy cattle body weight at different ages
Source: Genet Sel Evol. 2019 Feb 6;51:4. doi: 10.1186/s12711-018-0444-4 (PMC6366057; doi:10.1186/s12711-018-0444-4)
Supplement: Supplementary file 3 — Additional file 3. Potential candidate genes for maternal genetic effects on body weights recorded at birth (BW0), at 2 to 3 months of age (BW23) and at 13 to 14 months of age (BW1314). [file 12711_2018_444_MOESM3_ESM.docx]

**Additional file 3. Potential candidate genes for maternal genetic effects on body weights recorded at birth (BW0), at age month 2 to 3**

**(BW23) and at age month 13 to 14 (BW1314).**

| **Gene** | **Chr.** | **Start** | **End** | **BW0** | **BW23** | **BW1314** |
| --- | --- | --- | --- | --- | --- | --- |
| *SLC35B1* | 19 | 37546937 | 37553763 | X |  |  |
| *SPOP* | 19 | 37570928 | 37653473 | X |  |  |
| *NXPH3* | 19 | 37671531 | 37672235 | X |  |  |
| *NGFR* | 19 | 37725131 | 37744573 | X |  |  |
